# High-silica nanocrystalline Beta zeolites: efficient synthesis and catalytic application

**DOI:** 10.1039/c5sc03019f

**Published:** 2015-10-08

**Authors:** Raquel Martínez-Franco, Cecilia Paris, Marta E. Martínez-Armero, Cristina Martínez, Manuel Moliner, Avelino Corma

**Affiliations:** a Instituto de Tecnología Química (UPV-CSIC) , Universidad Politécnica de Valencia , Consejo Superior de Investigaciones Científicas , Valencia , 46022 , Spain . Email: acorma@itq.upv.es ; Email: mmoliner@itq.upv.es

## Abstract

An efficient synthesis methodology to obtain homogeneous nanosized high-silica Beta zeolites (∼10–20 nm) with high solid yields (above 95%) using simple alkyl-substituted flexible dicationic OSDAs is described. These dicationic OSDAs allow the synthesis of nanosized Beta zeolites with different Si/Al ratios (15–30) in alkaline and fluoride media, resulting in nanocrystalline materials with different physico-chemical properties. These nanosized Beta zeolites show better catalytic behavior towards the industrially-relevant alkylation of benzene with propylene to obtain cumene compared with other commercially available nanosized Beta zeolites.

## Abstract 2

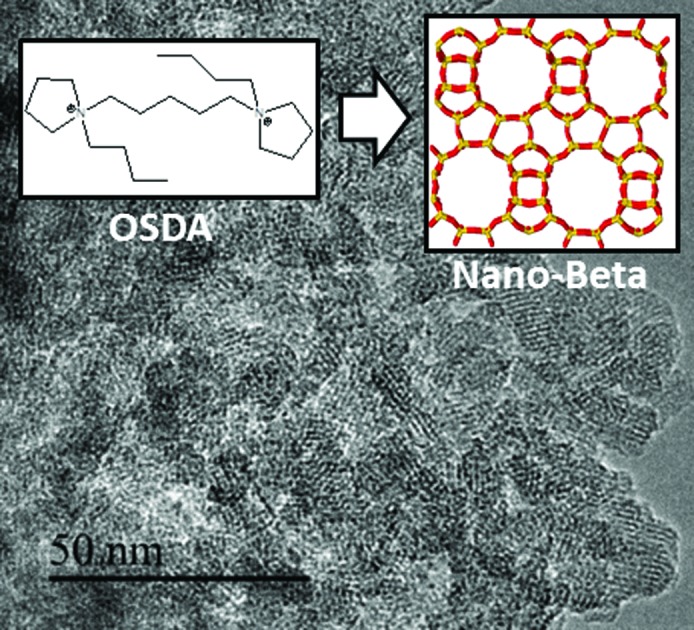
An efficient synthesis methodology to obtain homogeneous nanosized high-silica Beta zeolites (∼10–20 nm) with high solid yields (above 95%) using simple alkyl-substituted flexible dicationic OSDAs is described.

## 1. Introduction

The synthesis of nanosized zeolites has received significant attention in recent years, since the reduction of the crystalline particle size to the nanometer scale (below 100 nm) not only provides improved properties for traditional catalytic applications, but also allows the expansion of their use to other emerging applications, such as nanomedicine, optoelectronics, or chemical sensing.^1^

Beta is an industrially relevant zeolite with catalytic applications in petrochemistry,^2^ fine chemistry,^3^ biomass-transformations,^4^ and environmental chemistry.^5^ This broad applicability can be explained by the combination of a very open crystalline structure, formed of three-directional interconnected large pores,^6^ and the ability to be synthesized under very broad chemical compositions.^6^,^7^

The synthesis of nanocrystals of Beta zeolite that present large external surface area and low diffusion path lengths is of particular interest in order to improve the accessibility of reactants to the catalytic active sites.^1^ In this sense, nanocrystalline high-silica Beta zeolites with particle sizes below 100 nm have been described in the literature following different synthesis procedures. The first nanocrystalline high-silica Beta with crystal sizes in the 10 to 100 nm range was prepared by hydrothermal synthesis using tetraethylammonium hydroxide (TEAOH) as an organic structure directing agent (OSDA) in the absence of alkali metal cations.^8^ This methodology allows the synthesis of nanocrystalline Beta zeolites with different Si/Al ratios (from 6 to 50), but with a low solid yield (∼50%) for samples synthesized with Si/Al ratios above 10.^8^ Similar results have been described by other authors using TEAOH as an OSDA under related hydrothermal synthesis conditions.^9^ The synthesis of mesoporous high-silica Beta zeolites (Si/Al ∼ 8–30) formed by the assembly of uniform nanocrystals (20–100 nm) with high solid yields (∼80–90%) has also been described using TEAOH as the OSDA following steam-assisted conversion (SAC) procedures.^10^ However, SAC methodologies are difficult to scale-up for industrial applications. Recently, high-silica nanocrystalline Beta zeolites with intercrystalline mesoporosity have been described with good solid yields through hydrothermal synthesis methods,^11^ but they require the use of rigid and bulky organic compounds containing phenyl or biphenyl groups, such as 4,4′-trimethylenebis(*N*-methyl,*N*-benzyl-piperidinium),11*a* or 3,10-diazoniabicyclo[10.2.2]hexadeca-12,14,15-triene-3,3,10,10-tetramethyl-dichloride,11b,c and cationic polymers, such as poly diallyldimethylammonium chloride.11*d* Therefore, the efficient synthesis of high-silica nanocrystalline Beta zeolites in high-solid yield using simple OSDAs is still a challenging and relevant issue.

**Fig. 1:**
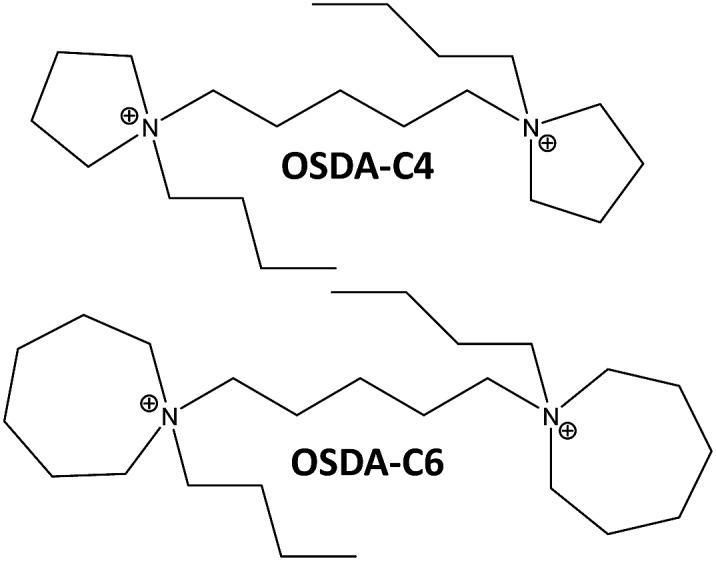
Organic structure directing agents used for the synthesis of nanosized Beta zeolites.

Here, we present an efficient synthesis procedure that affords nanosized high-silica Beta zeolites (∼10–20 nm) with solid yields above 95%, with simple alkyl-substituted flexible dicationic OSDAs (see Fig. 1) under hydrothermal synthesis conditions. Then, nanosized Beta zeolites with different Si/Al ratios (15–30) could be obtained with dicationic OSDAs in alkaline and fluoride media, and materials with different physico-chemical properties have been obtained. The catalytic behavior of the different nanosized Beta zeolites has been evaluated for an industrially-relevant chemical process, the alkylation of benzene with propylene to obtain cumene, revealing significantly improved catalytic activity compared to commercially available nanocrystalline Beta zeolites.

## 2. Experimental section

### 2.1. OSDA syntheses

#### Synthesis of the 1,1′-(pentane-1,5-diyl)bis(1-butylpyrrolidin-1-ium) [OSDA-C4]

In a two-neck round flask previously dried at 110 °C and equipped with a glass condenser, 1-butylpyrrolidine (38.17 g, 0.30 moles) was introduced and dissolved with 200 ml of anhydrous *N*,*N*-dimethylformamide (DMF). Then, 1,5-dibromopentane (22.99 g, 0.10 moles) was gradually added under vigorous stirring and an argon atmosphere. The reaction mixture was allowed to react at 90 °C overnight. Once the reaction was finished the crude product formed was decanted and separated from the reaction crude mixture by filtration at reduced pressure. The product was washed twice with ethyl acetate and once with diethyl ether to eliminate the remaining DMF. Finally, OSDA-C4 was obtained as a white solid after recrystallization with a mixture of 2-propanol/ethyl acetate with an 82.1% yield.

For its use in the synthesis of zeolites, the final product was ion exchanged to the hydroxide form using a commercially available hydroxide ion exchange resin (Dowex SBR).

#### Synthesis of the 1,1′-(pentane-1,5-diyl)bis(1-butylazepan-1-ium) [OSDA-C6]

##### Synthesis of 1-butylazepan-1-ium bromide

In a two-neck round flask connected with a glass condenser, 22 g of hexamethyleneimine (0.222 moles) was dissolved with 100 ml of anhydrous acetonitrile. Under vigorous magnetic stirring and an argon atmosphere, 30.40 g of 1-bromobutane (0.222 moles) was added dropwise over one hour. After the addition was finished, the mixture was heated at 70 °C and left to react for 8 hours. The molten salt was separated from the solution by filtration under reduced pressure and washed with ethyl acetate. The 1-butylazepan-1-ium bromide was obtained as a crystalline white solid with an 85.3% yield.

##### Synthesis of 1-butylazepane

Equimolar quantities of 1-butylazepan-1-ium bromide and sodium carbonate (0.187 moles), were introduced in a one-neck round flask and dissolved with distilled water. The resulting mixture was left to react for one hour at room temperature under magnetic stirring. A pale oily product was formed at the bottom of the flask. The organic phase was decanted and separated from the aqueous solution. The organic phase was dried with anhydrous magnesium sulphate salt, and then filtered to separate the inorganic salt. Finally, after elimination of solvent and purification, 1-butylazepane was obtained as a transparent oil with 77.4% yield.

##### Synthesis of 1,1′-(pentane-1,5-diyl)bis(1-butylazepan-1-ium) bromide (OSDA-C6)

The 1-butylazepane (22.36 g, 0.144 moles) obtained in the previous step was introduced in a two-neck round flask equipped with a glass condenser. 400 ml of anhydrous DMF was added to the flask under an argon flow. Then, 1,5-dibromopentane (16.56 g, 0.072 moles) was gradually added under vigorous stirring. After the addition was finished, the mixture was allowed to react overnight at 70 °C. A white precipitate was formed when the reaction mixture was cooled down to room temperature. The solid was isolated from the crude mixture by filtration at reduced pressure, washed twice with ethyl acetate to eliminate DMF and then dried. Finally, OSDA-C6 was obtained as a white solid after recrystallization with 2-propanol/ethyl acetate solution.

For its use in the synthesis of zeolites, the final product was ion exchanged to the hydroxide form using a commercially available hydroxide ion exchange resin (Dowex SBR).

### 2.2. Zeolite syntheses

In a typical synthesis, aluminum hydroxide [Al(OH)_3_, Sigma-Aldrich] was dissolved in an aqueous solution of the OSDA in its hydroxide form. Colloidal silica (Ludox AS-40, Aldrich) was then added, and the mixture was maintained under stirring for 20 minutes. If required, a 10% wt solution of NH_4_F (Sigma-Aldrich) was added, and the resultant mixture gel was allowed to reach the desired silica to water ratio by evaporation under stirring. The final gel compositions were: SiO_2_ : 0.0167–0.033Al_2_O_3_ : 0.2–0.4OSDA(OH)_2_: 0–0.4NH_4_F : 3–30H_2_O, where OSDA can be one of the dicationic molecules described above.

Finally, the gels were transferred to Teflon lined stainless autoclaves and heated at 150 °C for 10 days. The solids were recovered by filtration, extensively washed with distilled water, and dried at 90 °C overnight. The samples were calcined in air at 550 °C for 4 hours. The resultant solid yields have been calculated based on silica + alumina conversion.

### 2.3. Characterization

Powder X-ray diffraction (PXRD) measurements were performed with a multisample Philips X'Pert diffractometer equipped with a graphite monochromator, operating at 45 kV and 40 mA, and using Cu Kα radiation (*λ* = 0.1542 nm).

The chemical analyses were carried out on a Varian 715-ES ICP-Optical Emission spectrometer, after solid dissolution in HNO_3_/HCl/HF aqueous solution.

The morphology of the samples was studied with field emission scanning electron microscopy (FESEM) using a ZEISS Ultra-55 microscope and with field emission transmission electron microscopy (TEM) using a JEM 2100F microscope.

Textural properties were obtained from the N_2_ adsorption–desorption isotherms measured at 77 K with Micromeritics ASAP 2020 apparatus.

Solid NMR spectra were recorded at room temperature with a Bruker AV 400 MAS spectrometer. ^27^Al MAS NMR spectra were recorded at 104.2 MHz with a spinning rate of 10 kHz and a 9° pulse length of 0.5 μs with a 1 s repetition time. ^27^Al chemical shift was in reference to Al^3+^(H_2_O)_6_.

Infrared spectra were measured with a Nicolet 710 FT IR spectrometer. Pyridine adsorption–desorption experiments were made on self-supported wafers (10 mg cm^–1^) of original samples previously activated at 673 K and 10^–2^ Pa for 2 hours. After wafer activation, the base spectrum was recorded and pyridine vapor (6.5 × 10^2^ Pa) was admitted into the vacuum IR cell and adsorbed onto the zeolite. Desorption of pyridine was performed under vacuum over three consecutive one-hour periods of heating at 423, 523 and 623 K, each of them followed by an IR measurement at room temperature. The spectra were scaled according to the sample weight.

### 2.4. Catalytic test

Liquid phase alkylation of benzene with propylene was carried out with the acid zeolites, pelletized, crushed, and sieved at 0.25–0.42 mm diameter. The reaction was performed in an automated high pressure stainless steel reactor, at 3.5 MPa, 398 K, WHSV = 25 h^–1^ with a benzene to propylene (B/P) molar ratio of 3.5. The composition of the outlet stream was analyzed on-line on a Varian-450 gas chromatograph equipped with a 30 m 5% phenyl–95% dimethylpolysiloxante capillary column connected to a flame ionization detector. More details can be found in a previous study.^12^

## 3. Results and discussion

### 3.1. Synthesis and characterization of the nanosized Betas

In a preliminary experimental design under alkaline conditions using the OSDA-C4 molecule (see Fig. 1) as the OSDA, the influence of different synthesis variables, such as Si/Al (15, 30), OSDA/Si (0.2, 0.4) and H_2_O/Si (15, 30) molar ratios, have been explored at 150 °C with a 10 day synthesis time.

**Fig. 2:**
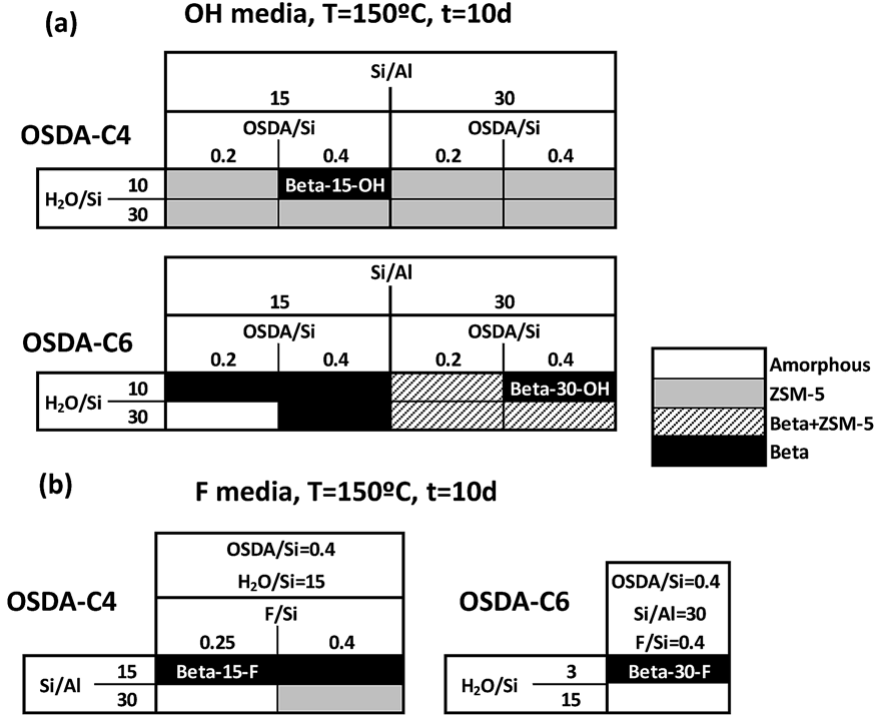
Phase diagrams obtained using OSDA-C4 and OSDA-C6 as OSDAs.

As seen in Fig. 2a, OSDA-C4 allows the preferential crystallization of ZSM-5 materials under most of the studied conditions, especially for high Si/Al ratios (∼30), and diluted gels (H_2_O/Si ∼ 30). Similar ZSM-5 materials have been recently reported by Burton under analogous synthesis conditions.^13^ Interestingly, Beta zeolite material was crystallized at Si/Al, OSDA/Si and H_2_O/Si molar ratios of 15, 0.4 and 10, respectively in the gel (see Beta-15-OH in Fig. 2a). The as-prepared Beta-15-OH zeolite shows a Si/Al ratio of 15.3 (see Table 1) and ^27^Al MAS NMR spectroscopy reveals that all the aluminum is tetrahedrally coordinated within the zeolitic framework (see Fig. 4a). Moreover, the solid yield obtained is above 95%, according to the initial oxide sources used in the synthesis.

**Table 1:** Chemical analyses of the as-prepared nanosized Beta zeolites

Sample	Si/Al
Beta-15-OH	15.3
Beta-30-OH	30.6
Beta-15-F	16.0
Beta-30-F	29.8
CP811	13.0

**Fig. 3:**
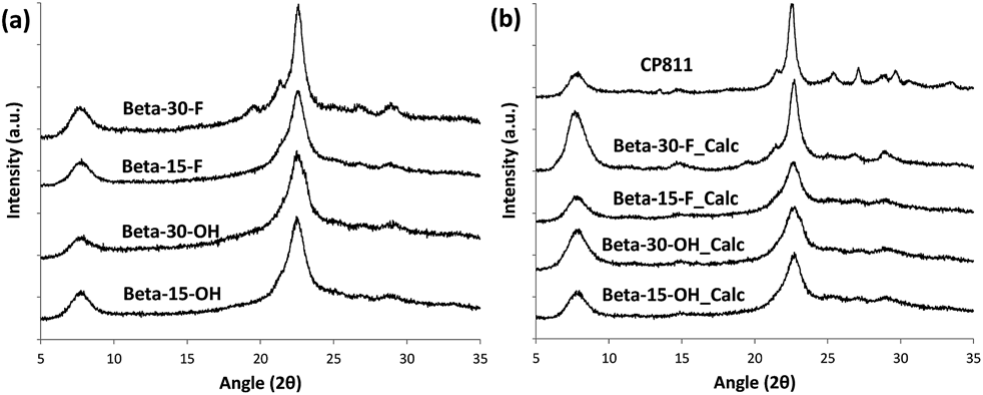
PXRD patterns of the as-prepared (a) and calcined (b) nanosized Beta zeolites.

**Fig. 4:**
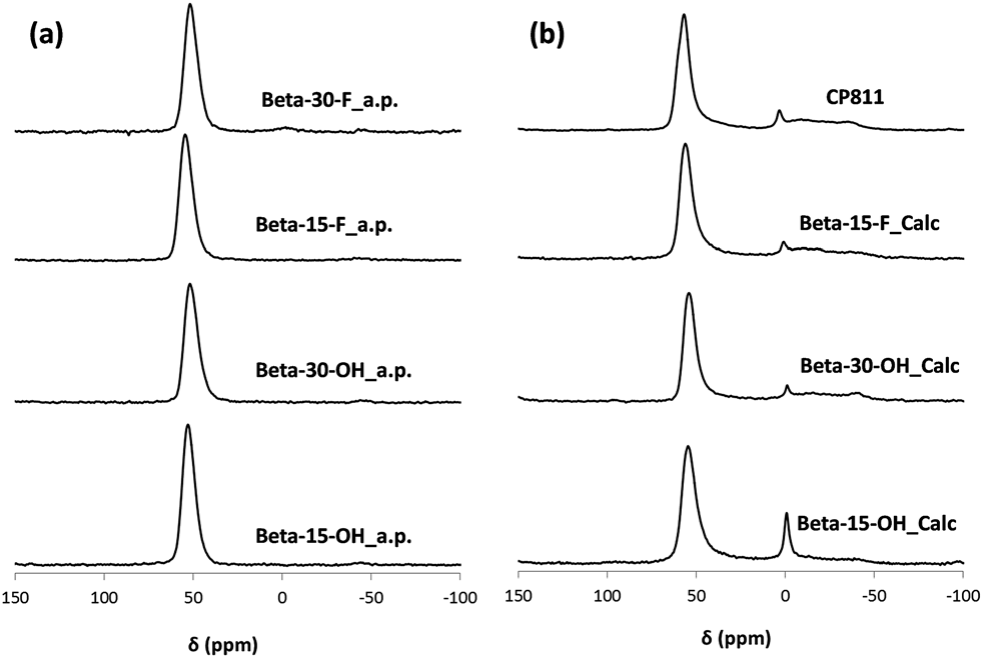
^27^Al MAS NMR spectra of the as-prepared (a) and calcined (b) nanosized Betas.

**Fig. 5:**
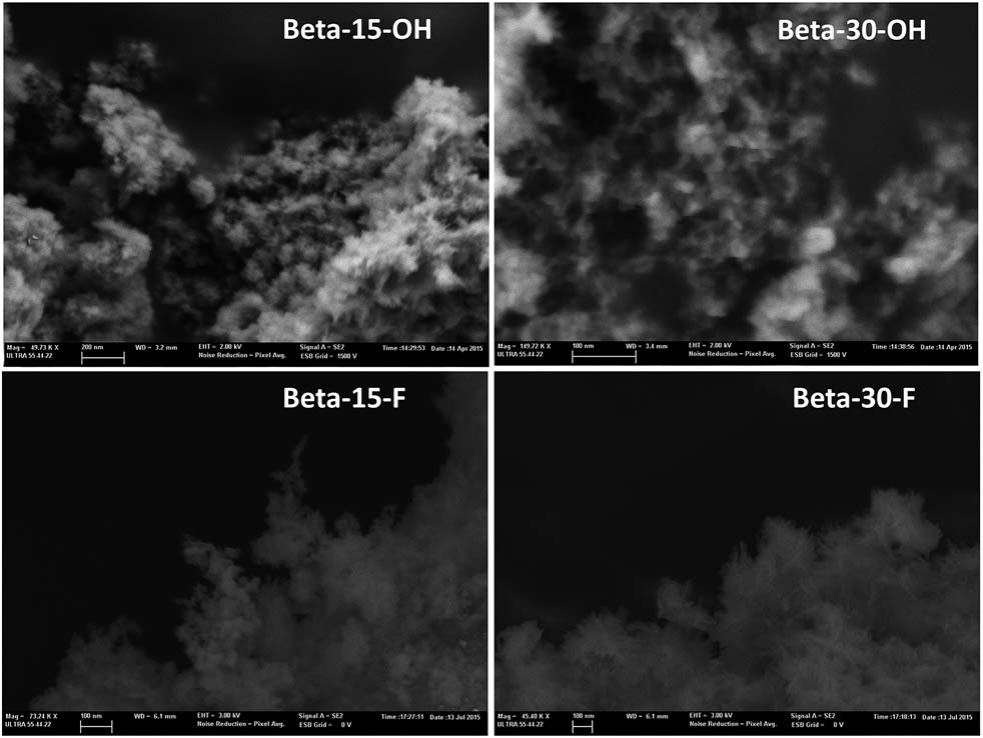
FE-SEM images of the as-prepared nanosized Beta zeolites.

**Fig. 6:**
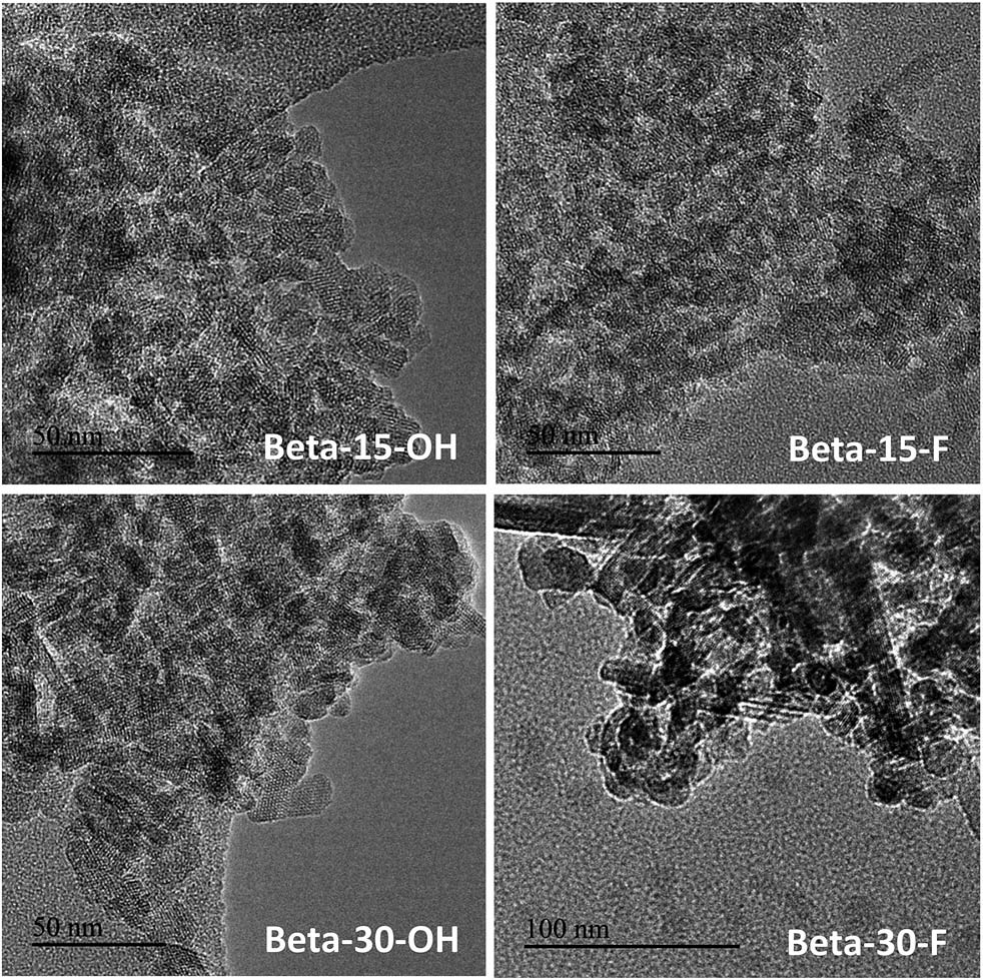
TEM images of the synthesized nanosized Beta zeolites.

The low-intense and broad diffraction peaks observed in the PXRD pattern of this Beta zeolite suggest that this material should be in the form of small crystallites (see Beta-15-OH in Fig. 3a). FE-SEM microscopy confirms the formation of very small homogeneous crystals (see Beta-15-OH in Fig. 5), and TEM microscopy reveals that the average particle size is ∼10 nm (see Beta-15-OH in Fig. 6).

**Fig. 7:**
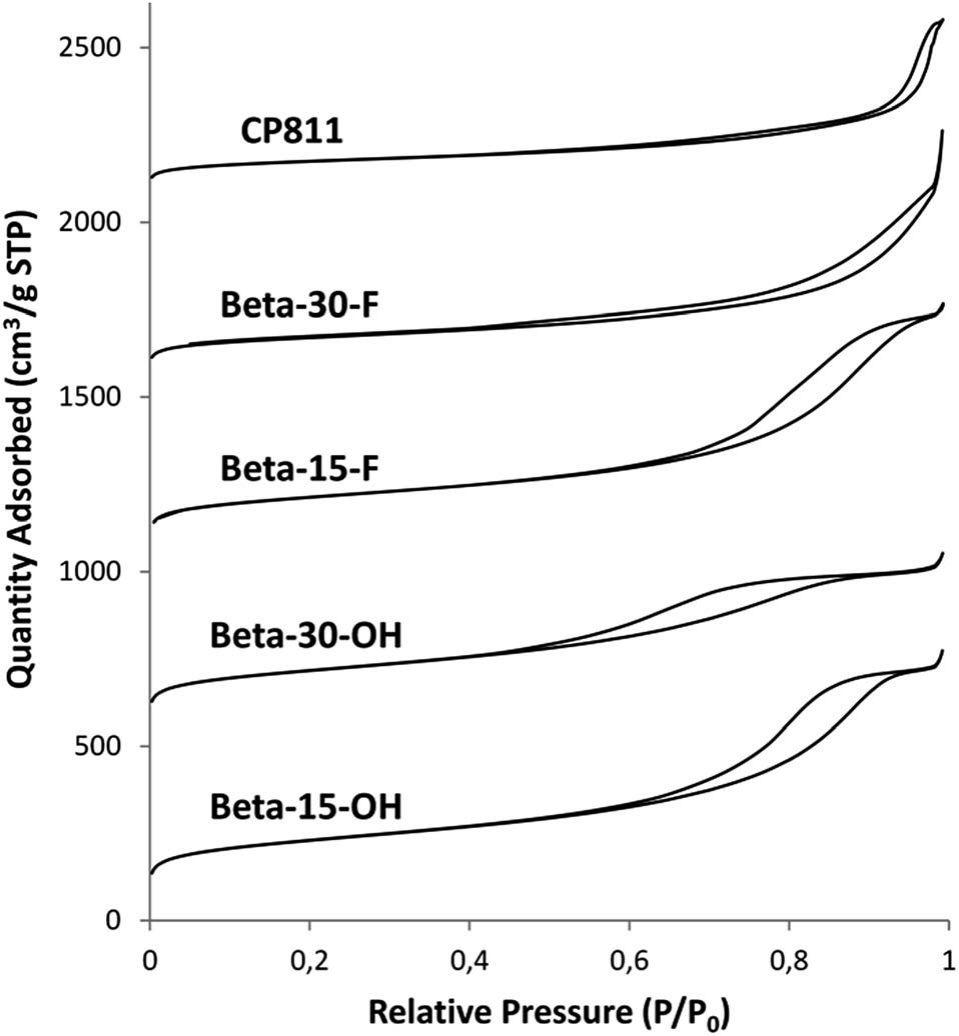
N_2_ adsorption isotherms of the nanosized Beta zeolites in their calcined form.

The N_2_ adsorption–desorption isotherms of Beta-15-OH (see Fig. 7) after calcining the sample at 550 °C in the presence of air show a steep rise uptake at low *P*/*P*_0_ pressure, which is typical for microporous materials, and an inflection and hysteresis loop at *P*/*P*_0_ between 0.6–0.9, which can be explained by a large interparticle capillary condensation due to the presence of small crystal sizes. The measured BET surface area is 757 m^2^ g^–1^, with an external surface area and a micropore volume of 440 m^2^ g^–1^ and 0.15 cm^3^ g^–1^, respectively (see Table 2).

**Table 2:** Textural properties of the nanosized Beta zeolites in their calcined form measured by N_2_ adsorption/desorption

Sample	Area BET (m^2^ g^–1^)	Ext. surface area (m^2^ g^–1^)	Micr. area (m^2^ g^–1^)	Micr. vol. (cm^3^ g^–1^)
Beta-15-OH	757.4	439.9	317.5	0.15
Beta-30-OH	738.2	428.7	309.5	0.14
Beta-15-F	719.8	396.2	323.6	0.15
Beta-30-F	568.5	241.1	327.4	0.16
CP811	580.0	203.1	378.4	0.18

**Fig. 8:**
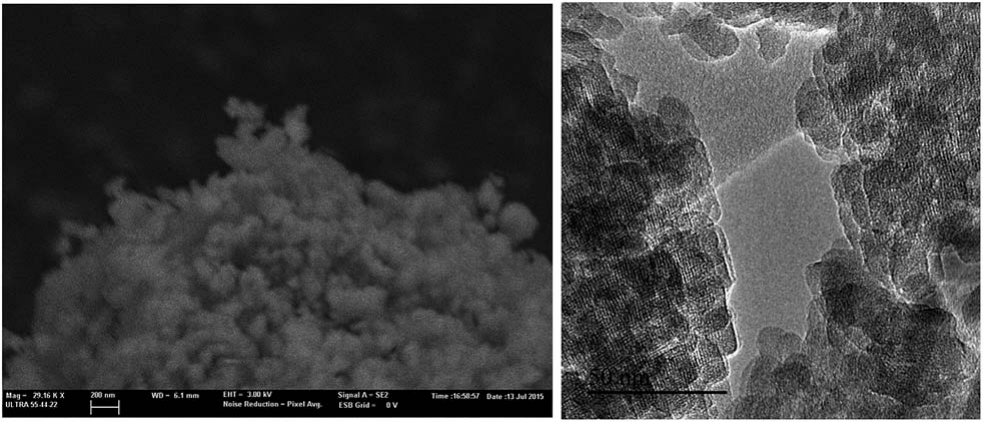
FE-SEM (left) and TEM (right) images of the commercial nanocrystalline Beta zeolite (CP811).

For comparison purposes, a commercially available nanocrystalline Beta zeolite (CP811, Zeolyst) with a similar Si/Al ratio (∼13) has also been characterized. This nanosized Beta zeolite shows larger particle sizes (∼20–25 nm, see TEM image for CP811 in Fig. 8) and lower BET and external surface areas (580 and 203 m^2^ g^–1^, respectively, see Table 2). Thus, the nanosized high-silica Beta zeolite with a Si/Al ratio of 15 prepared using OSDA-C4 (see Fig. 1) shows improved physico-chemical properties compared to the commercially available nanocrystalline Beta zeolite.

However, attempts to synthesize nanosized Beta zeolites with higher Si/Al ratios using OSDA-C4 as the OSDA resulted in the crystallization of ZSM-5 materials (see Fig. 2a).

To favor the nucleation and crystallization of the nanosized large pore Beta instead of the medium pore ZSM-5 zeolite at higher Si/Al ratios, we thought to use a dicationic OSDA molecule similar to OSDA-C4 but presenting larger heterocyclic end-groups (see OSDA-C6 in Fig. 1). We based this hypothesis on the fact that larger heterocyclic end-groups would hardly allow the nucleation of the medium pore ZSM-5 zeolite due to host–guest size constraints, favoring the crystallization of the large pore Beta zeolite. Thus, the OSDA-C6 molecule was tested as an OSDA under the same synthesis conditions used before for OSDA-C4. As can be seen in Fig. 2a, OSDA-C6 allows the preferential formation of Beta zeolite under a wider range of conditions, achieving the crystallization of a pure Beta zeolite with a Si/Al ratio of 30 in the synthesis medium (see Beta-30-OH in Fig. 2a). This as-prepared sample shows a Si/Al ratio of 30.6 in the final solid (see Table 1) and the ^27^Al MAS NMR spectrum only shows a band at ∼54 ppm, associated with aluminum in tetrahedral coordination (see Fig. 4a). The solid yield achieved is above 95%, based on the initial oxide sources.

As observed above for the Beta-15-OH sample, the PXRD pattern of Beta-30-OH also shows the presence of broad diffraction peaks, which are typical of zeolites with very small crystal sizes (see Fig. 3a). SEM and TEM images confirm the formation of nanosized Beta crystals, with an average size comprised between 10–15 nm (see Beta-30-OH in Fig. 5 and 6). In addition, N_2_ adsorption–desorption characterization shows a BET surface area of 738 m^2^ g^–1^, with an external surface area and a micropore volume of 428 m^2^ g^–1^ and 0.14 cm^3^ g^–1^, respectively (see Table 2).

The results obtained could be an indication that this type of dicationic OSDA would be the origin of the small crystallites obtained. If this was so, they may generate nanocrystalline Beta even during the synthesis in the presence of F^–^, where large crystal sized zeolites are most generally obtained. Then, the synthesis of high-silica nanosized Beta zeolites was attempted using dicationic OSDA-C4 and OSDA-C6 molecules under fluoride media (see Fig. 2b). As can be seen in Fig. 2b, the crystallization of the Beta zeolite in the presence of fluoride anions in the synthesis media could be accomplished with two different Si/Al ratios, 15 and 30 (see Beta-15-F and Beta-30-F in Fig. 2b). These as-prepared solids show similar Si/Al ratios to the ones introduced in the synthesis gels (16 and 29.8 for Beta-15-F and Beta-30-F, respectively, see Table 1), where all the aluminum is tetrahedrally coordinated within the zeolitic frameworks (see the ^27^Al MAS NMR spectra in Fig. 4a). The solid yields were also above 95% in both cases.

TEM images reveal crystallites with average particle sizes of 10 and 30–50 nm for Beta-15-F and Beta-30-F, respectively (see Fig. 6). In fact, the smaller crystal sizes observed for the Beta-15-F sample results in higher BET and external surface areas (720 and 396 m^2^ g^–1^, respectively, see Table 2) compared to Beta-30-F (568 and 241 m^2^ g^–1^, respectively, see Table 2). Notice that the materials obtained present much smaller crystallites than the reported synthesis of Beta in fluoride media with sizes within several microns.^14^

### 3.2. Catalytic application of the nanosized Betas

**Fig. 9:**
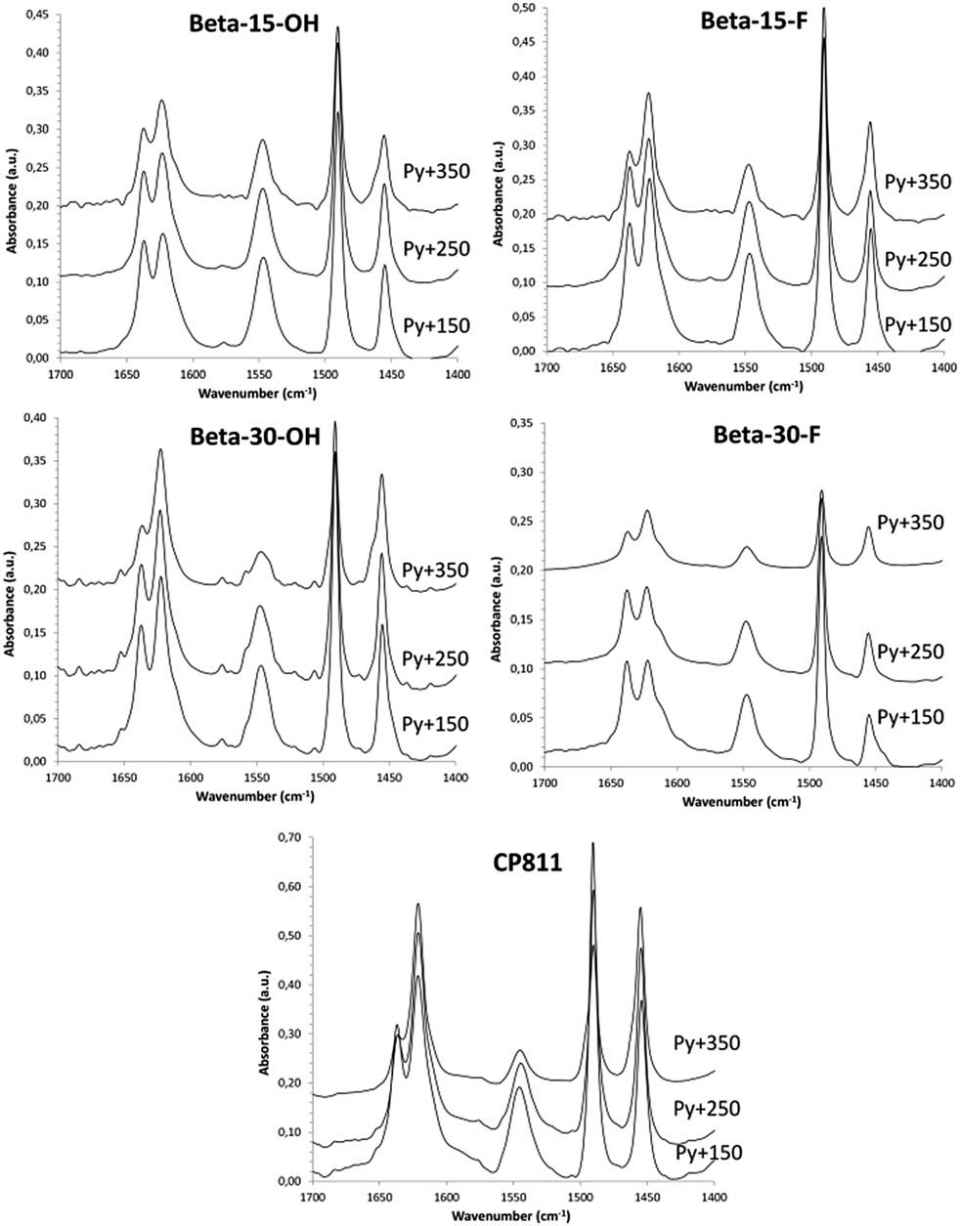
Transmission FTIR spectra in the stretching C–C region of the different nanosized Beta zeolites after adsorbing pyridine followed by desorption at 150, 250, and 350 °C.

The application of these materials as heterogeneous acid catalysts in industrially-relevant chemical processes is very important to evaluate their acid properties. Since Brønsted acidity is directly related to the amount of aluminum in framework positions, the stability of the aluminum species in the calcined nanosized Beta samples was studied by solid ^27^Al MAS NMR spectroscopy. As it can be seen in Fig. 4b, the ^27^Al MAS NMR spectra show that close to 80% of the aluminum species remain as Al^IV^ within the calcined nanocrystalline Beta zeolites. Notice that this percentage is similar for the small crystallite commercial CP811 material. The Brønsted acidity of these materials has been studied using *in situ* FTIR spectroscopy combined with the adsorption/desorption of pyridine. The FTIR spectra of adsorbed pyridine after desorption treatment at 150, 250, and 350 °C are represented in Fig. 9. As it can be seen in Fig. 9, all the nanosized Beta zeolites show the characteristic IR band of the pyridinium ion at 1545 cm^–1^, which is associated with the presence of Brønsted acid sites. Interestingly, the IR band of the pyridinium ion is mostly retained after increasing the desorption temperature to 350 °C, revealing the presence of strong Brønsted acidities (see Fig. 9). The amount of Brønsted and Lewis acid sites per gram of zeolite can be determined from the IR bands centered at 1545 and 1455 cm^–1^, respectively.^15^ The nanocrystalline Beta zeolites synthesized with a Si/Al ratio of 15, both Beta-15-OH and Beta-15-F, show the highest amount of Brønsted acid sites together with the commercial CP811 material (see B150 in Table 3), with a remarkably large amount of pyridine retained after desorption treatment at 350 °C in these materials (∼90 μmol pyr per g, see B350 in Table 3). As expected, the two nanosized Beta zeolites synthesized with a Si/Al ratio of 30 show lower Brønsted acidity, corresponding to their lower Al content (see Table 3).

**Table 3:** Acidity of nanosized Beta zeolites as determined by FT-IR combined with pyridine adsorption–desorption

Sample	Acidity (μmol pyr per g)					
	B150	B250	B350	L150	L250	L350
Beta-15-OH	146	130	94	68	64	52
Beta-30-OH	110	98	47	84	82	71
Beta-15-F	163	144	88	87	75	71
Beta-30-F	71	53	23	24	21	19
CP811	220	165	85	211	205	193

**Fig. 10:**
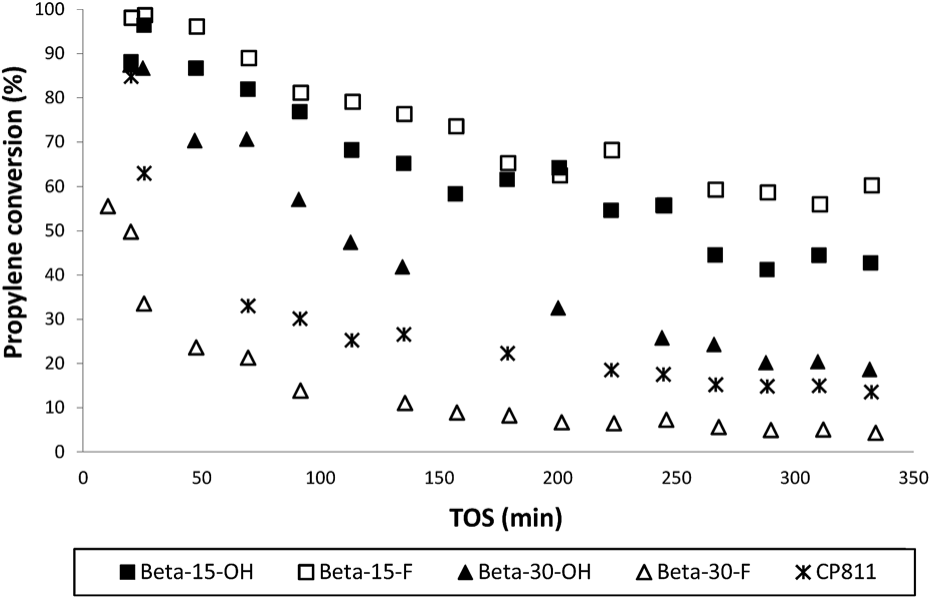
Propylene conversion with TOS for the liquid phase alkylation of benzene with propylene using nanosized Betas as catalysts.

**Fig. 11:**
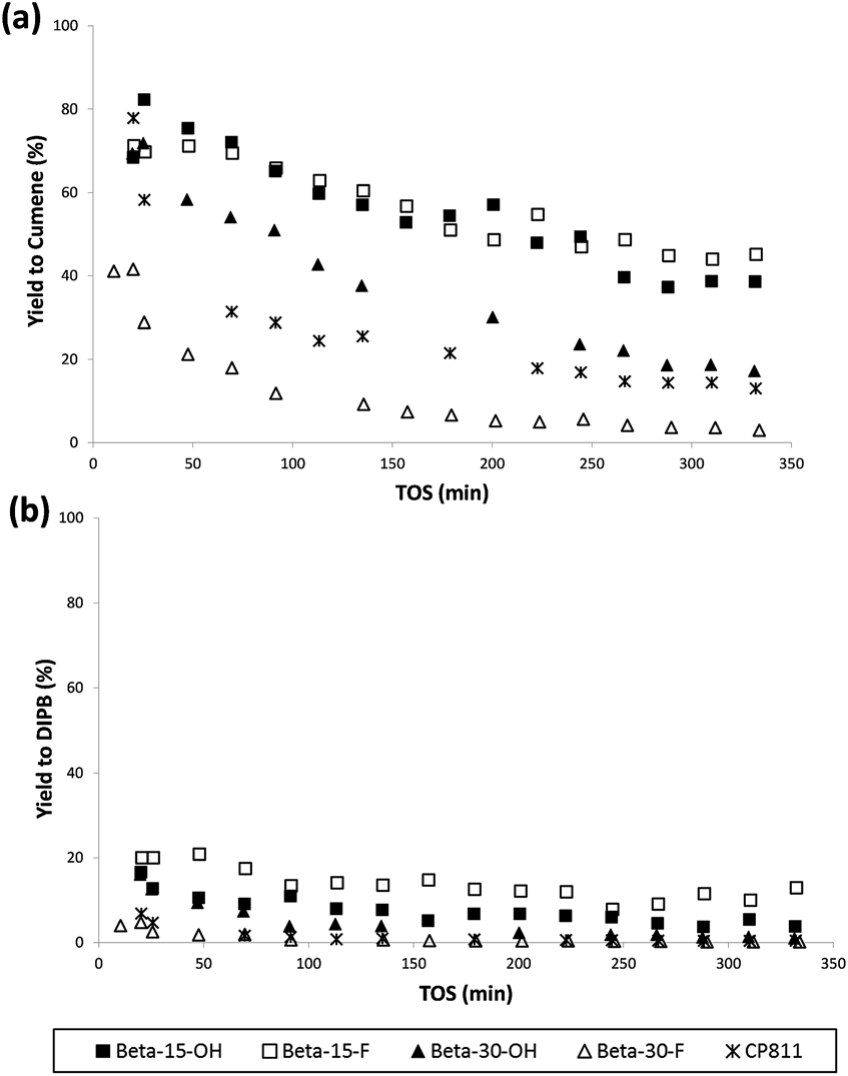
Selectivity to cumene (a) and di-isopropylbenzene (b) with TOS for the liquid phase alkylation of benzene with propylene using nanosized Betas as catalysts.

The catalytic activity of these nanosized Beta zeolites has been studied for the alkylation of benzene with propylene to produce cumene, which is an important industrial intermediate product to obtain phenol and acetone.^16^ This industrial process is mainly operated in the liquid phase using large pore zeolites as heterogeneous acid catalysts.^16^ However, the design of large pore acid zeolites presenting nanosized crystals is convenient for this catalytic process, to reduce the restriction of cumene diffusion and to decrease the catalyst deactivation by olefin oligomerization and pore occlusion by multi-alkylated subproducts. As can be seen in Fig. 10, the two nanosized Beta zeolites with a Si/Al ratio of 15, Beta-15-OH and Beta-15-F, not only show better initial catalytic activity as compared to the commercial nanocrystalline Beta zeolite (CP811) despite the lower acidities of the former, but also a remarkable decrease of the catalyst deactivation with time on stream (TOS). In addition, the yield to the desired cumene is also significantly higher for Beta-15-OH and Beta-15-F zeolites (see Fig. 11a).

With respect to the samples synthesized with a Si/Al ratio of 30, the Beta-30-OH shows an intermediate catalytic activity between Beta-15-F and the commercial CP811 zeolite (see Fig. 10 and 11). In this sense, the lowest catalytic activity of Beta-30-OH could be explained by its lower Brønsted acidity compared to Beta-15-F (see Table 3), but its high external surface area decreases the catalyst deactivation compared to CP811 (see Table 2 and Fig. 10).

## 4. Conclusions

In summary, we have presented an efficient synthesis to achieve nanosized Beta zeolites with particle sizes comprised between 10 and 20 nm. This has been obtained with two different non-surfactant alkyl-substituted flexible dicationic OSDAs that allow the synthesis of nanocrystalline Beta zeolites under broad Si/Al ratios (15–30) in alkaline and fluoride media with excellent solid yields (above 95%). These nanosized Beta zeolites show better catalytic behavior to obtain cumene compared to a commercially available nanosized Beta zeolite for the alkylation of benzene with propylene.
